# Intraoperative Label-Free Multimodal Nonlinear Optical Imaging for Point-of-Procedure Cancer Diagnostics

**DOI:** 10.1109/jstqe.2021.3054578

**Published:** 2021-01-26

**Authors:** Lingxiao Yang, Jaena Park, Marina Marjanovic, Eric J. Chaney, Darold R. Spillman, Heidi Phillips, Stephen A. Boppart

**Affiliations:** Beckman Institute for Advanced Science and Technology, University of Illinois at Urbana-Champaign, Urbana, IL 61801 USA; Small Animal Surgery, Veterinary Teaching Hospital, University of Illinois College of Veterinary Medicine, Urbana, IL 61802 USA; Beckman Institute for Advanced Science and Technology, University of Illinois at Urbana-Champaign, Urbana, IL 61801 USA

**Keywords:** Nonlinear optical imaging, label-free, cancer imaging, intraoperative optical imaging, multiphoton microscopy, auto-fluorescence, multi-harmonic generation, needle biopsy, cancer diagnostics

## Abstract

Intraoperative imaging in surgical oncology can provide information about the tumor microenvironment as well as information about the tumor margin. Visualizing microstructural features and molecular and functional dynamics may provide important diagnostic and prognostic information, especially when obtained in real-time at the point-of-procedure. A majority of current intraoperative optical techniques are based on the use of the labels, such as fluorescent dyes. However, these exogenous agents disrupt the natural microenvironment, perturb biological processes, and alter the endogenous optical signatures that cells and the microenvironment can provide. Portable nonlinear imaging systems have enabled intraoperative imaging for real-time detection and diagnosis of tissue. We review the development of a label-free multimodal nonlinear optical imaging technique that was adapted into a portable imaging system for intraoperative optical assessment of resected human breast tissue. New developments have applied this technology to assessing needle-biopsy specimens. Needle-biopsy procedures most always precede surgical resection and serve as the first sampling of suspicious masses for diagnosis. We demonstrate the diagnostic feasibility of imaging core needle-biopsy specimens during veterinary cancer surgeries. This intraoperative label-free multimodal nonlinear optical imaging technique can potentially provide a powerful tool to assist in cancer diagnosis at the point-of-procedure.

## I. Introduction

CANCER is one of the leading causes of death in both humans [1]–[3] and companion animals [4], [5]. For cancer diagnosis, histopathology has been the gold standard for assessing surgical specimens and making diagnostic decisions [6]–[8]. However, conventional histological procedures require tissue fixation, sectioning, and staining [7], [9], which is both time- and labor-intensive. To accelerate the process by which a cancer diagnosis is made, various optical imaging techniques [10]–[27] have been developed to provide real-time histological assessment. Among them, tissue staining has been a widely-adopted procedure before imaging, especially in many fluorescence microscopy techniques [10]–[15]. Since exogenous dyes, stains, and nanoparticles can perturb the tissue microenvironment and disrupt biological processes, label-free optical imaging is of great importance in terms of its ability to provide biological and thus diagnostic information from intact and unperturbed tissue samples, even *in vivo*.

Previous research studies using optical coherence tomography (OCT) have demonstrated its ability to visualize sub-surface tissue structures in real-time without labeling, and this modality has enabled a variety of clinical applications, including intraoperative assessment of both the *in vivo* tumor resection bed as well as *ex vivo* surgical specimens [16]–[23]. More recently, label-free nonlinear optical imaging (NLOI) has become an active area of investigative research with growing biomedical applications. Label-free nonlinear optical imaging (NLOI) enables visualization of the microstructures, metabolic profiles, and even cellular dynamics in untreated biological tissue, especially important for cancer research [28]–[47]. Several label-free NLOI modalities, such as coherent anti-Stokes Raman scattering (CARS) microscopy [28], [29], stimulated Raman scattering (SRS) microscopy [30], [31], second harmonic generation (SHG) [32]–[38], third harmonic generation (THG) [39], [40], two-photon fluorescence (2PF) [41]–[50], and three-photon fluorescence (3PF) microscopy [51], [52] have been demonstrated in multiple biomedical studies. CARS and SRS are well-known techniques for biochemical visualization and quantification, especially for lipid and protein contents [28]–[31], while SHG has been widely used for visualizing collagen in biological specimens, producing robust signals with sub-micron resolution [32]–[35]. Many studies have shown the ability of these modalities to provide diagnostic information, as well as their potential to be an intraoperative imaging tool in various clinical applications [31], [35]–[37]. While the SHG signal vanishes in centrosymmetric materials [32], [53], THG has no limitations in material properties and is enhanced at interfaces between different refractive indices [54], [55], providing visualization of optical heterogeneities [39]. 2PF is another well-known label-free NLOI modality that enables deep tissue imaging [41], [42], and its development has fostered the identification and applications of various endogenous fluorophores such as flavin adenine dinucleotide (FAD) and the reduced nicotinamide adenine dinucleotide (NAD(P)H) [43]–[49]. FAD and NAD(P)H have been identified as natural indicators for metabolic activity [45]–[47] and used for non-invasive label-free metabolic imaging [43]–[49]. 3PF has further pushed the imaging depth limit in biological tissues and has been demonstrated in *in vivo* brain imaging [48], [49].

With the unique visualization capabilities of each modality, which are also based on different physical mechanisms, the integration of multiple modalities can provide more comprehensive information about biological samples. This multimodal imaging therefore can represent both the microstructures and metabolic profiles of cells and tissues, and do this rapidly in real-time to capture cell and tissue dynamics. Our Biophotonics Imaging Laboratory has developed many label-free multimodal NLOI techniques for simultaneous multi-contrast imaging [57]–[67], including 2PF, 3PF, SHG, THG, and CARS. 2PF and 3PF signals give the distribution of FAD and NAD(P)H, respectively. SHG highlights collagen fibers, and THG reveals the boundaries of various important biological components such as cells and lipid droplets. CARS at 2850 cm^−1^ and 3050 cm^−1^ correspond to the CH_2_ and CH aromatic stretching, which indicates the relative lipid and protein content, respectively [57]–[59]. These combined nonlinear modalities can provide label-free molecular profiling of intact tissue specimens, which is becoming increasingly important for cancer diagnosis [57]–[67].

**Fig. 1.**
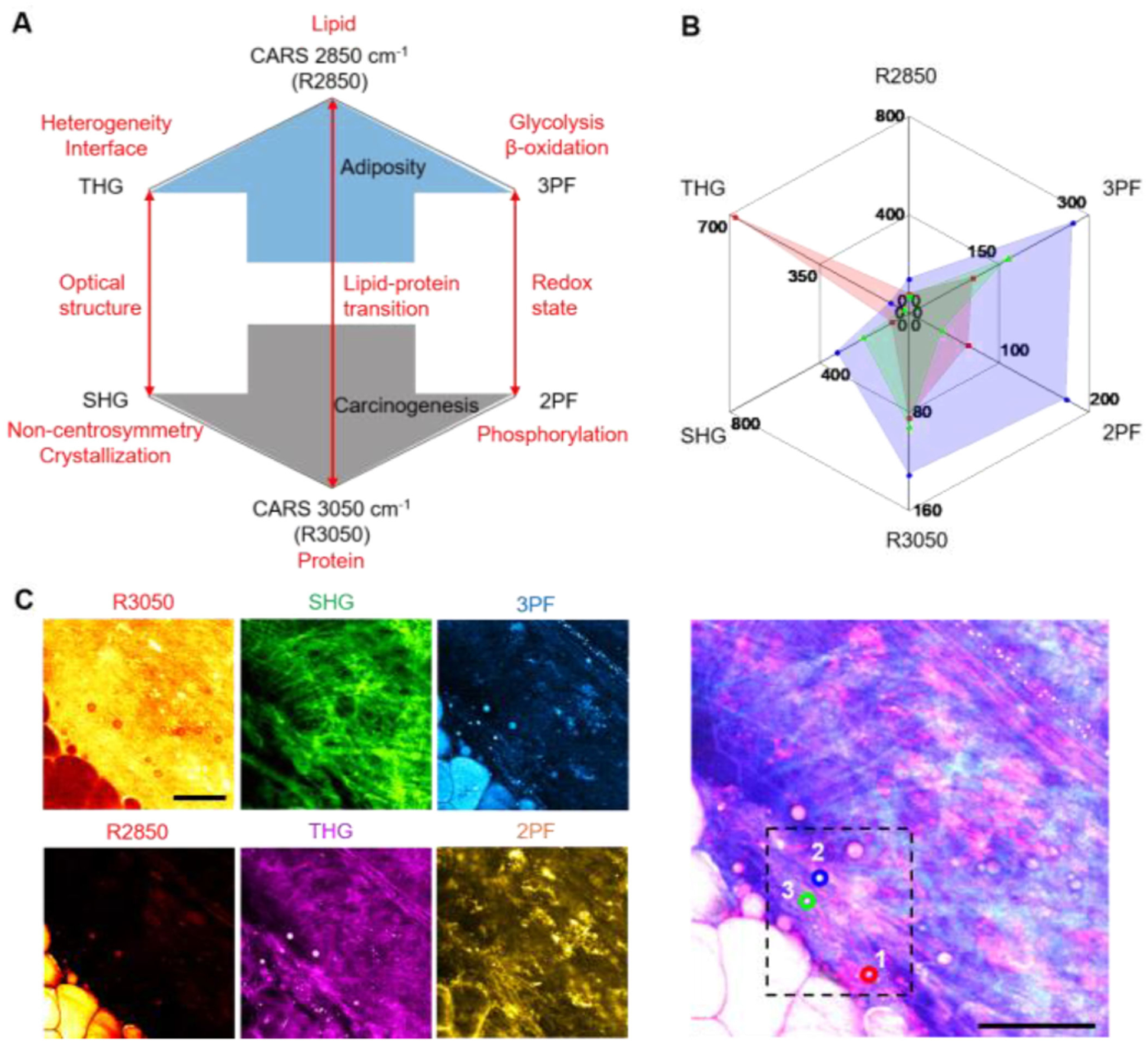
Molecular profiling based on label-free multimodal NLOI. (A) The design of the molecular profiling radar plot which relates the lipid-protein transition, the optical structures, and the redox state (red arrows) in the tissue and tumor microenvironments. The blue and grey arrows indicate the relative collective trend toward adiposity (normal) versus and carcinogenesis, respectively. (B) A representative example of molecular profiling using a radar plot generated from average pixel intensities of each modality over the selected regions of interest (ROI) in the dashed box in (C). Each color in the radar plot presents a different ROI. (C) Multimodal NLOI of rat mammary tumor development (6^th^ week in a longitudinal series). Right, composite 6-channel image with ROIs marked as labeled circles (1, red; 2, blue; 3, green), which correspond to the color-shaded regions in the radar plot in (B); Left, individual channel images: CARS (R3050 and R2850), coherent anti-Stokes Raman scattering; THG, third harmonic generation; 3PF, three-photon fluorescence; SHG, second harmonic generation; 2PF, two-photon fluorescence Scale bar represents 50 *μ*m. Modified figures used with permission from [59].

Lipid-protein transitions, optical structures, and metabolic signatures can be viewed graphically to constitute a unique molecular profile, as shown in Fig. 1A. Fig. 1B shows a representative molecular profiling radar plot based on the multimodal imaging results shown in Fig. 1C for a rat mammary tumor and microenvironment in the 6^th^ week of tumor development [59]. The six NLOI modalities, including 2PF, 3PF, SHG, THG, and CARS at 2850 cm^−1^ (R2850) and 3050 cm^−1^ (R3050), provide images that reveal tumorigenic signatures, such as tumorigenic stromal cells and angiogenesis-associated 3PF-sensitive microvesicles near a developing vessel [59]. Plotting the average intensities of the multiple modalities from the selected regions of interest into a radar plot offers their visual graphical correlations, which could contribute to better understanding of the associated changes during carcinogenesis.

**Fig. 2.**
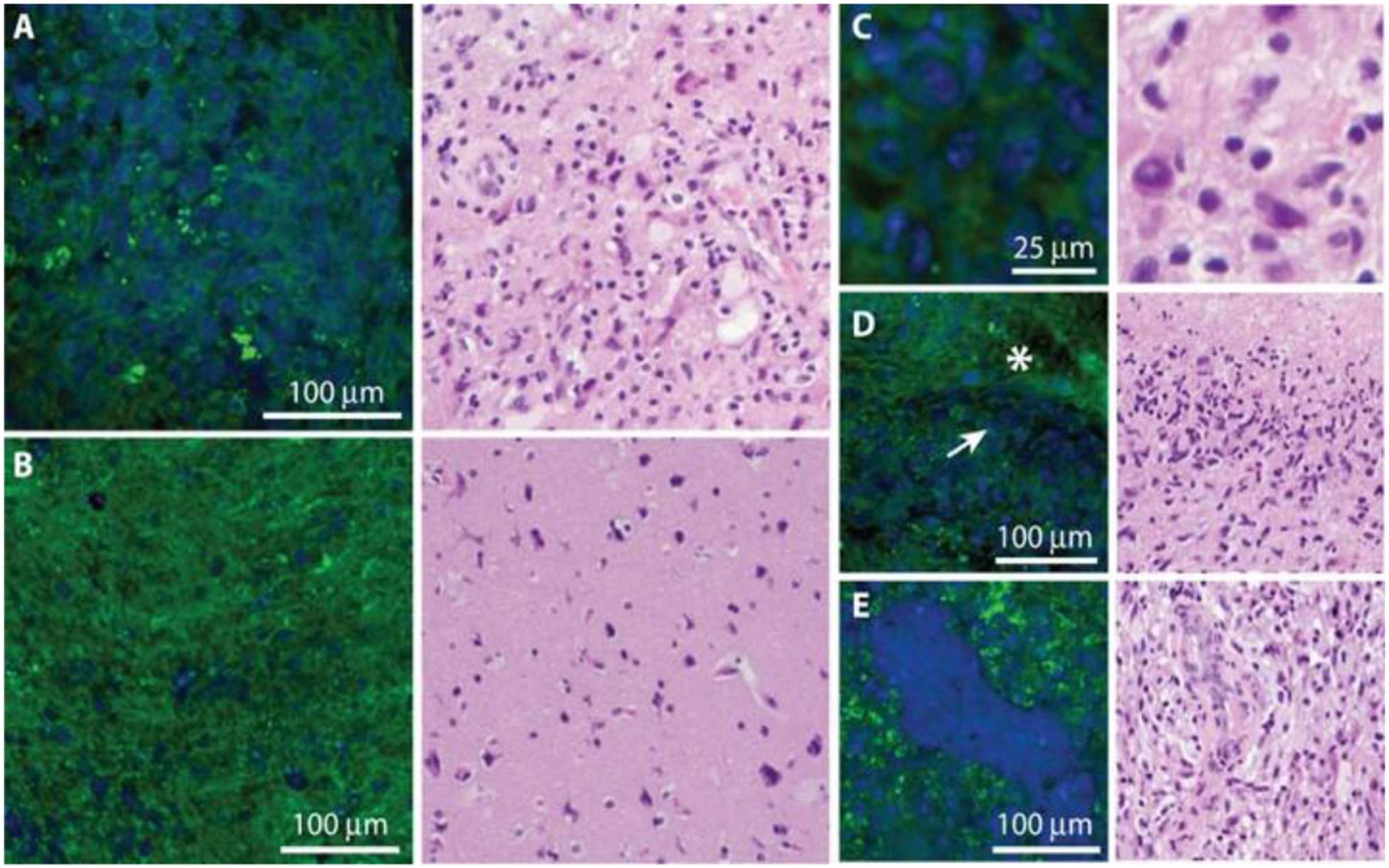
SRS microscopy of freshly excised human brain tumor specimens and corresponding H&E histology. (A) Images of viable tumor regions of show hypercellularity, in contrast with (B) normocellular regions of adjacent brain with minimal tumor infiltration. (C)–(E) Higher-magnification images of key diagnostic features of glioblastoma including cellular pleomorphism (C), pseudo-palisading necrosis, where densely cellular regions (arrow) border bland, acellular regions of necrosis (asterisk) (D), and microvascular proliferation (E). Proteins are false-colored in blue and lipids in green. Figure reprinted with permission from [31].

While SRS was not integrated into this multimodal platform, it is also an important label-free NLOI technique that has recently been applied to cancer diagnoses. Research has shown its ability to differentiate between tumor and non-tumor regions in freshly excised human brain tissue, as shown in Fig. 2A, B [31]. False-colored blue and green regions indicate signal from proteins and lipids, respectively. In the SRS images, the blue regions suggest hypercellularity due to the infiltration of tumor cells, while the green regions relate to the extracellular matrix, white matter tracts, or parenchyma in the brain [31]. Characteristic structures of glioblastoma have been identified and well-correlated with histology results, such as cellular pleomorphism (Fig. 2C), pseudo-palisading necrosis (Fig. 2D), and microvascular proliferation (Fig. 2E) [31]. However, since SRS relies on existing Raman bands for delineating tumors from normal tissues with sufficient signal-to-noise ratio (SNR), its ability to reveal structural and biochemical information is somewhat limited [31]. This suggests that a multimodal platform for label-free NLOI might be advantageous, not only for the advancement in fundamental cancer research, but also for intraoperative cancer imaging.

## II. Optical Imaging of Needle-Biopsy Specimens

Most intraoperative NLOI studies have involved imaging freshly excised surgical specimens, including the tumor, tumor margin, and adjacent normal tissue [27], [66]. However, prior to surgical intervention, a diagnosis of cancer is usually made via needle-biopsy (NB) with subsequent tissue fixation, sectioning, and staining. As with intraoperative imaging, there is also an essential need for real-time imaging and diagnosis during NB, at the point-of-procedure. As radiologic detection of small lesions with x-ray imaging and magnetic resonance imaging (MRI) has improved, it has become increasingly challenging to biopsy small lesions with needle-biopsy devices, even with image-guidance, and particularly for lesions deep within organs. Therefore, in addition to a real-time diagnostic technique, there is also a strong need simply to determine or “screen” NB-sampled tissue to determine if it contains abnormal tissue and ensure that the tissue will be diagnostically useful. We have further pursued the application of NLOI for both tissue specimen screening and diagnosis.

Needle biopsy has been used as a standard clinical outpatient procedure prior to more invasive surgery. With NB, small amounts of fluid or tissue are taken from suspicious areas and passed to pathologists for analysis and diagnosis [68]–[74]. NB procedures include: 1) fine-needle aspiration, where higher gauge (smaller diameter) needles are used to collect fluid and suspended cells for cytologic analysis, and 2) core-needle biopsies, where lower gauge (larger diameter) needles are used to retrieve cylindrical cores of tissue with preserved tissue architecture for histopathologic analysis. Compared to surgical resection, NB is less invasive with a smaller incision and targeted removal of the lesion. Imaging-guidance, such as with ultrasound imaging, MRI, x-ray imaging, or stereotactic x-ray imaging, is often used to identify the suspicious region for needle guidance, placement, and sampling. For the purposes of this review and research, we will focus on core-needle biopsy procedures for the diagnosis of cancer.

Many clinical studies of various types of cancers [69]–[74] have shown that the typical sensitivity of NB for differentiating tumors from normal tissue ranges from 82%–99%, which implies accuracy and thus the reliability of NB for retrieving diagnostically useful specimens. However, there is acknowledgement and concern about the time-consuming assessment of NB specimens. The conventional evaluation method, just as with excisional tissue biopsies, requires tissue fixation, sectioning, and staining, as mentioned previously. It often takes days to generate a complete pathological report [68]–[70]. Inadequate or non-diagnostic sampling can range from 5% to 34%, depending on tissue type and needle gauge [70], in which case additional NBs are required, further leading to diagnostic delay and increased anxiety and stress for the patient. Standard histological processing is also a destructive process, and results in the loss of functional biological information from the tissue, such as metabolic and molecular dynamics, which limits a more comprehensive understanding of cancer.

**Fig. 3.**
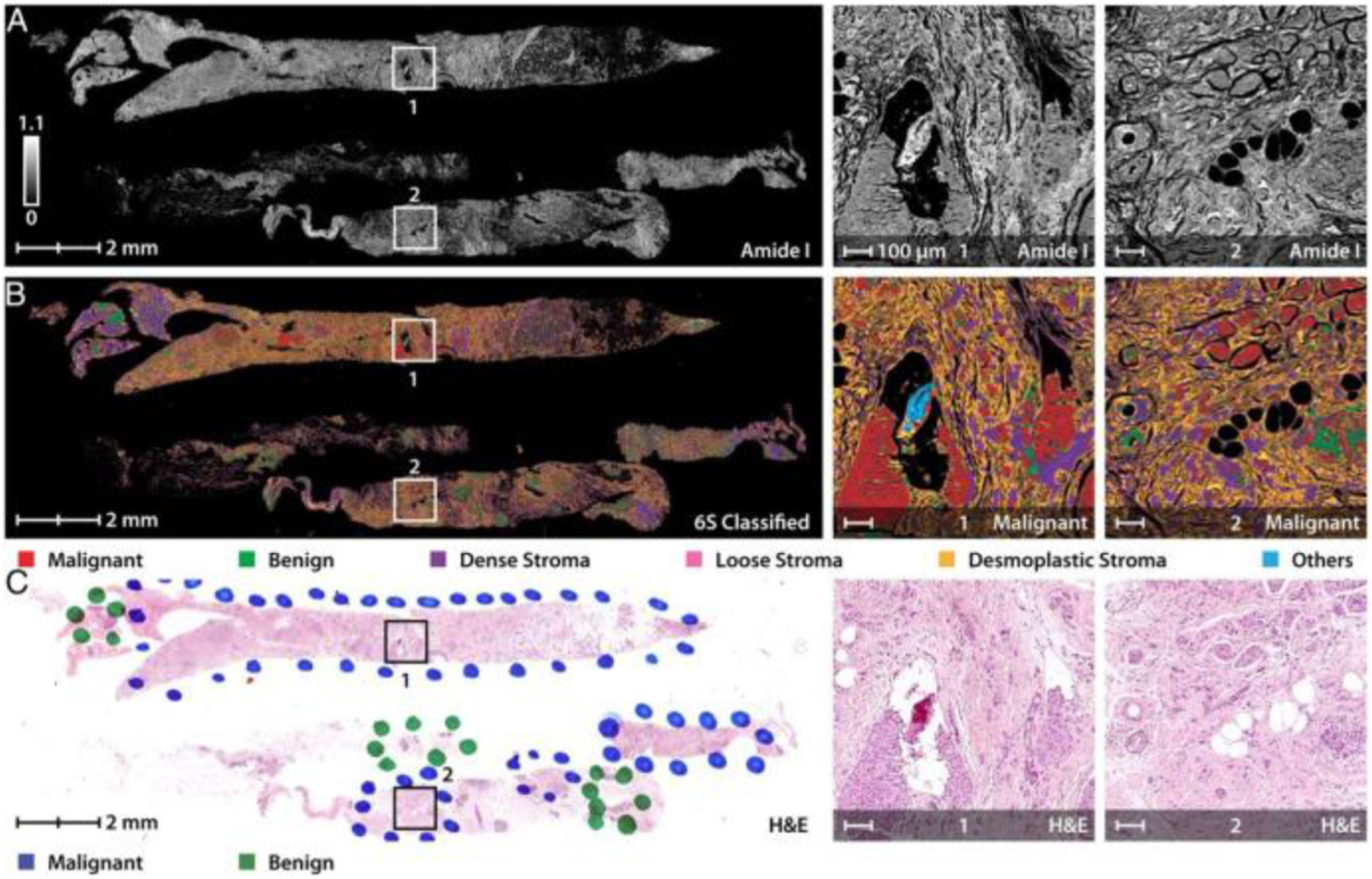
Rapid triaging of malignant sections using stain-free IR-imaging of human breast NB specimens. (A) Image of core needle biopsy sections at an absorbance frequency of 1658 cm^−1^ with specific malignant regions enlarged to the right for clarity. (B) Multi-spectral IR-image further classified using a 6-class model which distinguishes the cancerous and normal epithelial cells from various collagen-rich stromal types. (C) Pathologist annotations on the corresponding H&E stained histology section which is well-correlated to the IR-classified image. Figure reprinted with permission from [24].

To rapidly screen NB specimens for the presence of disease, and to shorten the overall time for NB evaluation, a few studies have demonstrated the potential of using different optical imaging modalities as an alternative to histopathology, such as confocal fluorescence microscopy [75], OCT [76], SHG [77], and infrared (IR)-imaging [24]. However, each carries various limitations, such as with transverse resolution and specificity [76], or complex and invasive staining procedures [75], [77]. Fig. 3 shows an example of rapid histological visualization of human breast core NB specimens using IR-imaging [24]. Although imaging of these core NB specimens in this work was stain-free, the tissue was fixed and paraffin-embedded before imaging. In addition, no metabolic or functional information could be retrieved from the imaging results, which has been increasingly important in cancer research. Therefore, to rapidly assess core NB specimens for the presence of abnormal features, to potentially enable a real-time initial diagnosis, and to gain a more comprehensive understanding of both the structural and functional changes occurring within core NB specimens, a label-free point-of-procedure evaluation method for biopsy specimens is needed.

## III. Label-Free Multimodal Nonlinear Optical Imaging

**Fig. 4.**
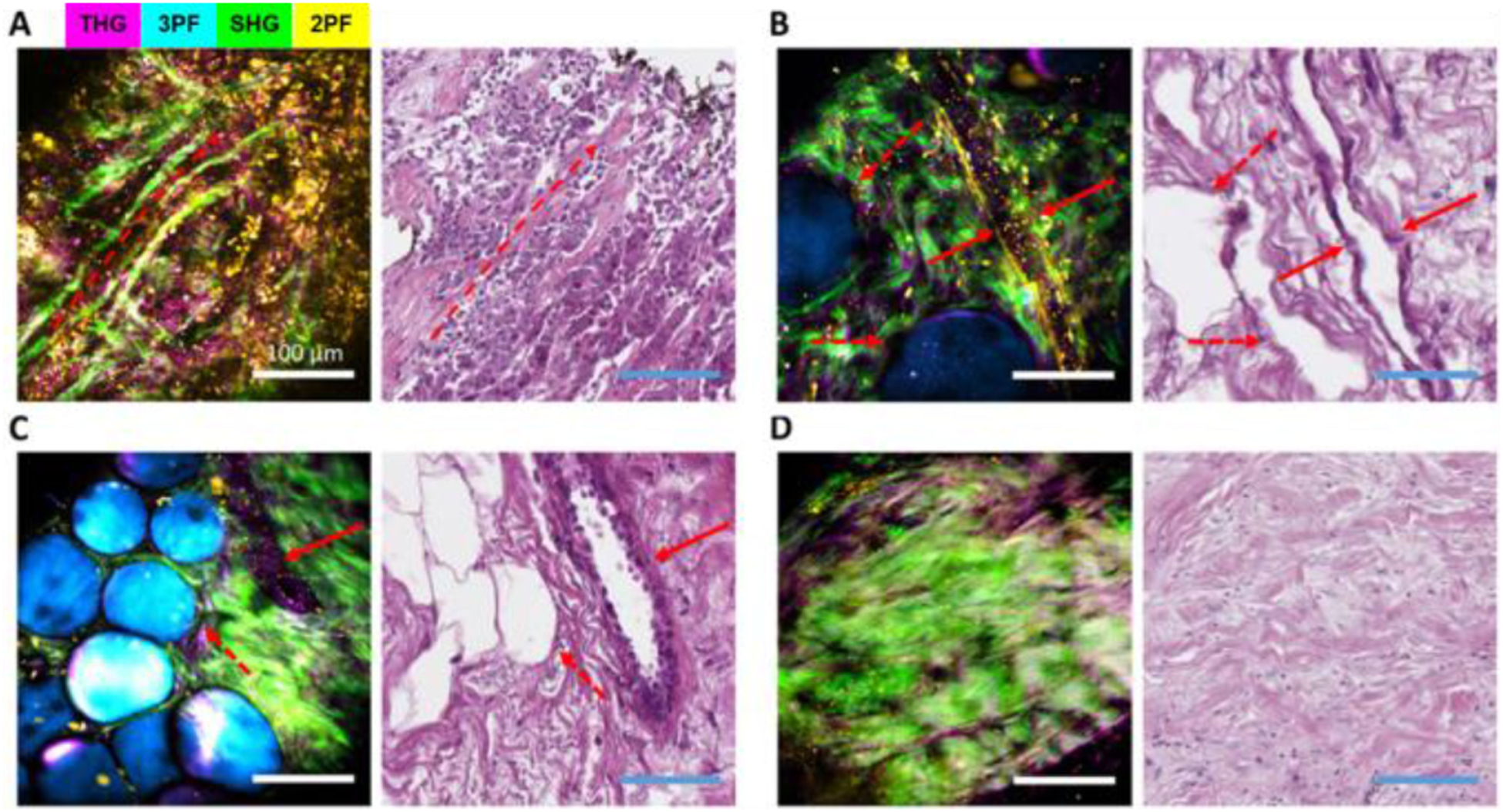
Label-free multimodal NLOI results (left) along with corresponding histology images (right) from human breast tissue specimens diagnosed as (A) invasive ductal carcinoma (IDC), with the red dashed arrow showing an overall orientation of collagen alignment, (B)–(C) ductal carcinoma *in situ* (DCIS), showing adipocytes (red dashed arrows), blood vessel (red solid arrow in (B)), and mammary duct (red solid arrow in (C)), and (D) healthy breast tissue from a breast reduction surgery. Scale bars represent 100 *μ*m. Channel pseudo-colors indicated by the legend in (A). Figure reprinted with permission from [66].

Multimodal NLOI platforms developed in the Biophotonics Imaging Laboratory have been designed and optimized to address the needs for capturing not only microstructural features, but also metabolic and dynamic cellular changes in biopsied tissue specimens. A bench-top simultaneous label-free autofluorescence multi-harmonic (SLAM) microscope was first demonstrated for intravital imaging in a carcinogen-induced rat mammary tumor model [60], demonstrating the ability for label-free multimodal NLOI to visualize cellular and extracellular structures in the unperturbed tumor microenvironment with high spatial and temporal resolutions, contrast, SNR, and speed [60]. To translate this novel technique to clinical applications, we subsequently customized and constructed a first-generation portable multimodal NLOI system [66]. A detailed comparison between the lab-based bench-top system and the clinical intraoperative system is discussed in the next section. Intraoperative imaging of freshly excised and unstained human breast tissue from breast cancer surgeries was performed to demonstrate the ability of this portable system for visualizing and characterizing the tumor site of invasive ductal carcinoma (IDC), featuring tumor cell infiltration along the well-aligned collagen fibers (Fig. 4A). The cell features are highlighted by the yellow (2PF) and magenta (THG) colors, where the 2PF signals originated from FAD content in the cells and the THG was generated from membranes [66]. The red dashed arrow shows the overall orientation of the collagen fibers (in green) from the SHG signal. Other structures in the tumor microenvironment, such as mammary ducts and blood vessels, are shown in the images obtained from a resected lumpectomy specimen with ductal carcinoma *in situ* (DCIS) (Fig. 4B, C). The blood vessel indicated by the solid red arrow in Fig. 4B is likely associated with angiogenesis. Small clusters of tumor cells can be found in the epithelium of the duct (Fig. 4C), highlighted by strong THG signals at their boundaries, 2PF signal associated with FAD content, and adipocytes that give rise to a strong 3PF signal from NAD(P)H [66]. A comparison with the normal breast tissue obtained during a breast reduction surgery from a subject with no history of cancer is shown in Fig. 4D, verified by the corresponding histology, where the collagen fibers appear to be curly and dense with no tumor cell infiltration or proliferation.

**Fig. 5.**
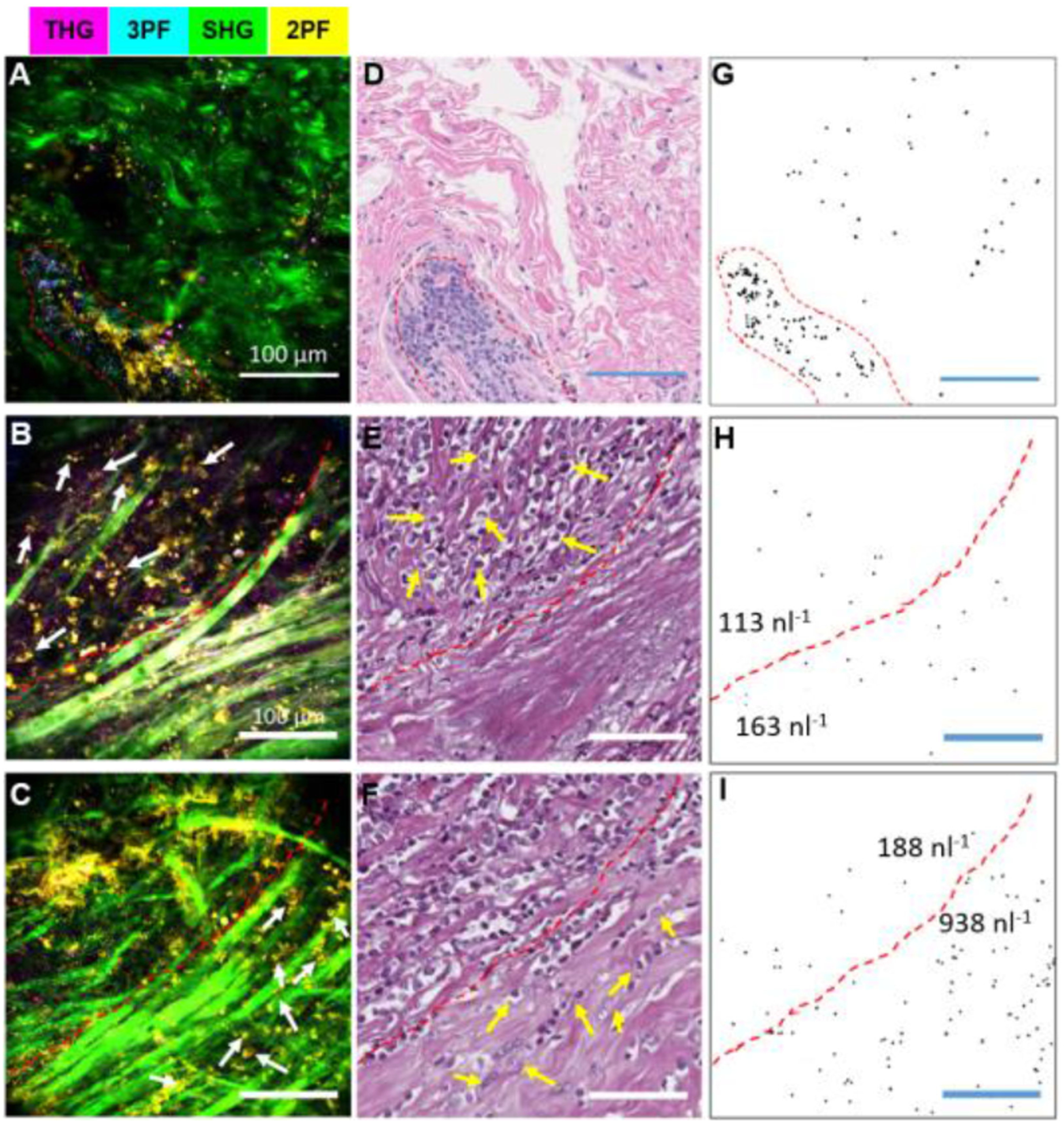
Spatial imaging of EV distribution and quantification in the tumor microenvironment. (A)–(C) Label-free multimodal NLOI of human breast tissue specimens with tumor-associated EVs. (D)–(F) Corresponding H&E-stained histology results. (G)–(I) Thresholded binary images of segmented EVs from the THG channel in the multimodal images (A)–(C), respectively. (A), (D), (G) EV enrichment in a region (enclosed by the red dashed line) of DCIS. (B), (E), (H) EVs in a boundary region (white arrows indicating tumor cells) of early-stage desmoplasia. (C), (F), (I) EVs in a region of later-stage desmoplasia with tumor-cell invasion. Note that the red dashed lines in (B), (C), (E), (F), (H), (I) indicate the interface or boundary between the tumor regions and the apparently normal collagen-rich regions. At the early stage of desmoplasia (H), the average EV density was found to be 144 nl^−1^, and EVs were approximately evenly distributed on each side of the tumor interface. In contrast, at a later stage (I), the average EV density increased to 575 nl^−1^ with a significantly higher level (938 nl^−1^) within the collagen-rich region, indicating an up-production of EVs along this interface of invasion. Scale bars represent 100 *μ*m. The color scale is the same as in Fig. 4. Figure reprinted with permission from [66].

In addition, these SLAM-based NLOI imaging systems have uniquely revealed the spatial distribution of extracellular vesicles (EVs) within the tumor microenvironment. Cancer-associated EVs have been recognized as a potential biomarker for cancer-related tissue changes, but previous analyses of EVs have traditionally been performed on dissociated cells or body fluids, with subsequent loss of spatial information and dynamics; important cues into their roles in carcinogenesis. The biodistribution and quantification of EVs were investigated in both preclinical animal models [62] and untreated human breast tissue specimens immediately after surgical excision [66]. Fig. 5 shows representative SLAM images (left column), along with corresponding H&E-stained histology (middle column), of desmoplasia in human breast tissue, as well as the identification and quantification of cancer-associated EVs from the SLAM images (right column). The binary images in the right column were generated from the THG channel, which was highly associated with the EVs. Fig. 5A, D, G show the EV enrichment in the DCIS region (enclosed by the red dashed line), supporting that tumor-associated EVs could be a unique biomarker for cancer [78]. Furthermore, comparing the EV distribution in the cases of early (Fig. 5B, E, H) and later stage desmoplasia (Fig. 5C, F, I), differences in EV density distribution can be seen, suggesting a potential means for tumor staging based on the spatial distribution of EVs in images, something that is not possible with current EV analysis methods from dissociated cells. This study implies that intraoperative label-free multimodal NLOI has the potential to provide histopathological assessment in real-time and at the point-of-procedure, and thus assist with cancer detection and diagnosis.

**Fig. 6.**
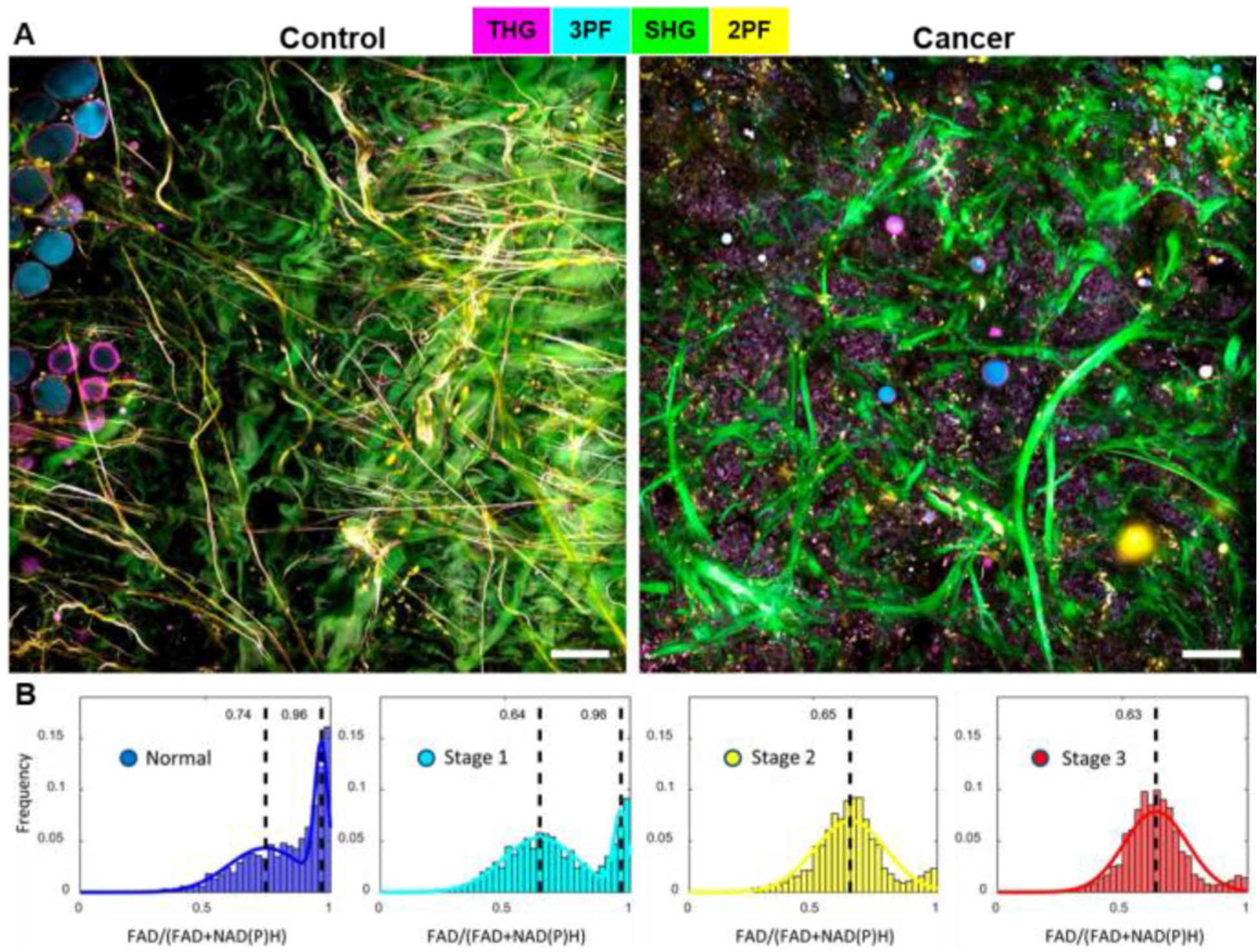
Label-free multimodal NLOI offers metabolic profiling of EVs as new biomarkers for real-time point-of-procedure cancer staging. (A) Label-free SLAM imaging of freshly excised normal and cancerous human breast tissues. (B) Histograms of optical redox values of EVs from subjects with different cancer stages (19 subjects in total, Normal [n = 7], Stage 1 [n = 5], Stage 2 [n = 3], and Stage 3 [n = 4]). FAD, flavin adenine dinucleotide; NAD(P)H, reduced nicotinamide adenine dinucleotide. The color scale is the same as in Fig. 4. Scale bar represents 100 *μ*m. Figure modified and reprinted with permission from [62].

The same SLAM-based technology and system was used for metabolic profiling of the visualized EVs in the tissue microenvironment. Fig. 6A shows representative label-free images of normal and cancer human breast tissue freshly excised during the surgeries. We adopted the established definition of the redox ratio as: FAD/(FAD+NAD(P)H) using the intensity of 2PF/(2PF+3PF) [48], [49]. Metabolic profiles of EVs from patients with different cancer stages (normal, stage 1 to stage 3) revealed statistically significant differences (Fig. 6B). EVs from later-stage cancer patients displayed a more Gaussian-like metabolic profile with a lower mean redox ratio value, which is mainly attributed to the increased concentration and percentage of the NAD(P)H-rich EVs associated with the more metabolically active cancer cells. This work demonstrated the ability of our label-free multimodal NLOI platform to provide metabolic information as part of the optical signatures for cancer staging and progression [62]. *In vivo* NLOI was also demonstrated in our previous work [60], [62] showing the ability of SLAM system to capture dynamic events (e.g., cell migration) in the unperturbed tissue microenvironment, which can contribute to better understandings of cancer despite the potential artifacts from animal respiratory and cardiac motion.

**Fig. 7.**
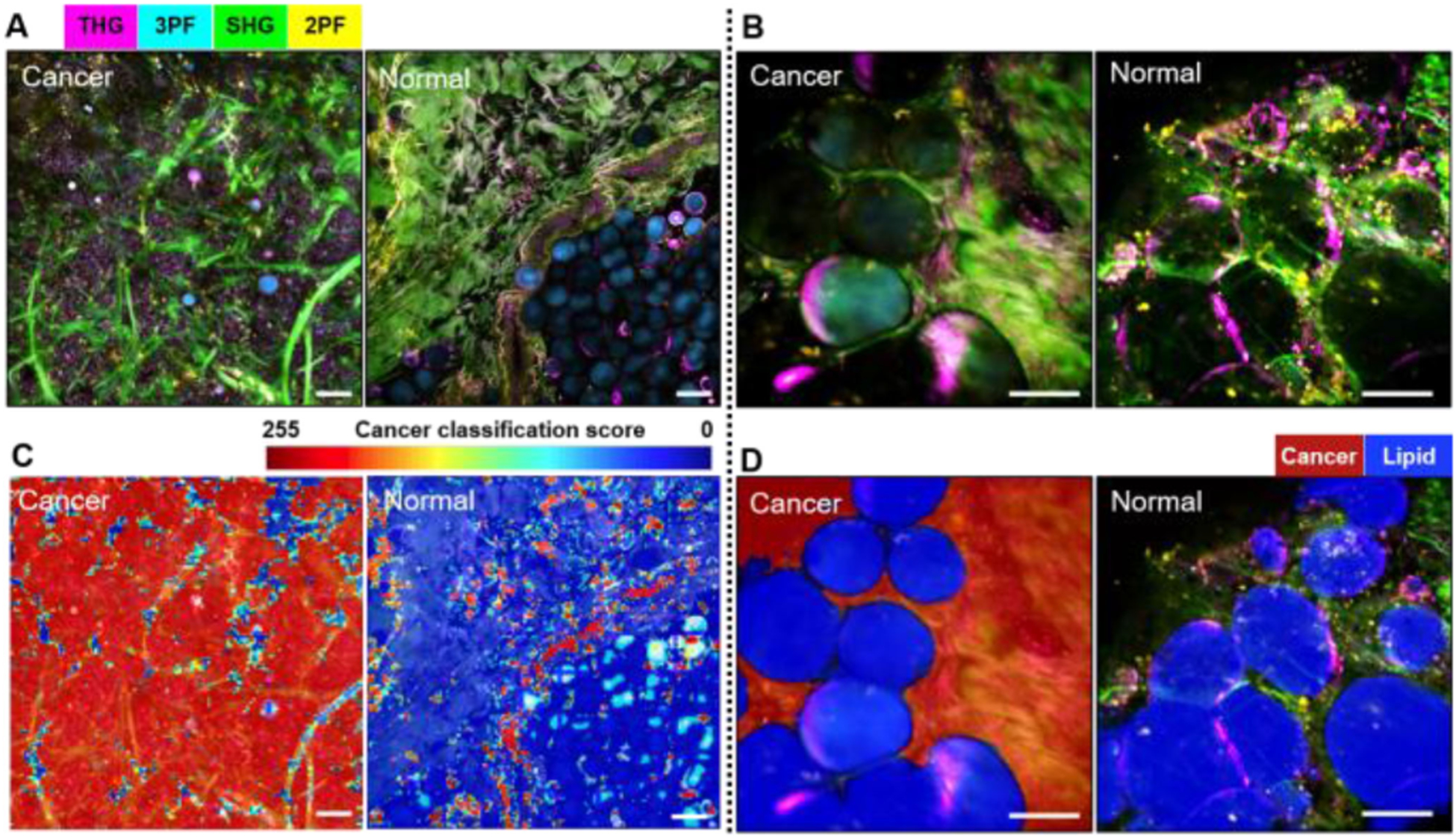
Label-free multimodal NLOI with artificial intelligence and deep-learning classification results. Left panels: (A) SLAM (lab-based) imaging of cancerous (left) and normal (right) human breast tissue along with corresponding classification maps (C). Right panels: (B) Intraoperative label-free multimodal NLOI of cancerous (left) and normal (right) human breast tissue along with corresponding classification results (D). Red indicates high-likelihood cancerous regions and blue is associated with higher lipid content. THG, third-harmonic generation; SHG, second harmonic generation; FAD, flavin adenine dinucleotide; NAD(P)H, reduced nicotinamide adenine dinucleotide. The color scale is shown on the top of (A). Scale bars represent 100 *μ*m. Figure modified and reprinted with permission from [67].

Most recently, with the development of artificial intelligence (AI), we have combined deep learning (DL) with our label-free multimodal NLOI techniques to enable real-time intraoperative virtual histology for cancer diagnosis [67]. The high dimensional multimodal image-based data sets generated by these NLOI systems provide a wealth of features, textures, patterns, and objects for AI/DL analysis. A custom DL framework has been designed for automatic cancer classification. The model was trained and optimized on the data sets acquired by the lab-based bench-top NLOI system (SLAM microscope). A discriminative localization method, Classification Activation Mapping [79], was then applied for detailed visualization of the cancer classification by the model. Fig. 7 shows SLAM images of cancer and normal human breast tissue (Figs. 7A, B), with the corresponding classification maps in Figs. 7C, D, respectively. It can be observed that the collagen fibers (green, SHG) and lipid content (cyan, 3PF) are less associated with cancer, while the tumor cells, tumor-associated EVs, altered blood vessels and mammary ducts are features that are more indicative of cancer [67]. The classification ability of the DL model was also tested on intraoperative NLOI data sets of cancer and normal breast tissue [67]. The combination of high-dimensional label-free multimodal NLOI and an AI/DL framework offers the potential for automated quantitative cancer detection, classification, and diagnosis that could be performed in real-time and at the point-of-procedure.

## IV. Translation of A Multimodal Nonlinear Optical Imaging System for Point-of-Care Needle Biopsy Assessment

**Fig. 8.**
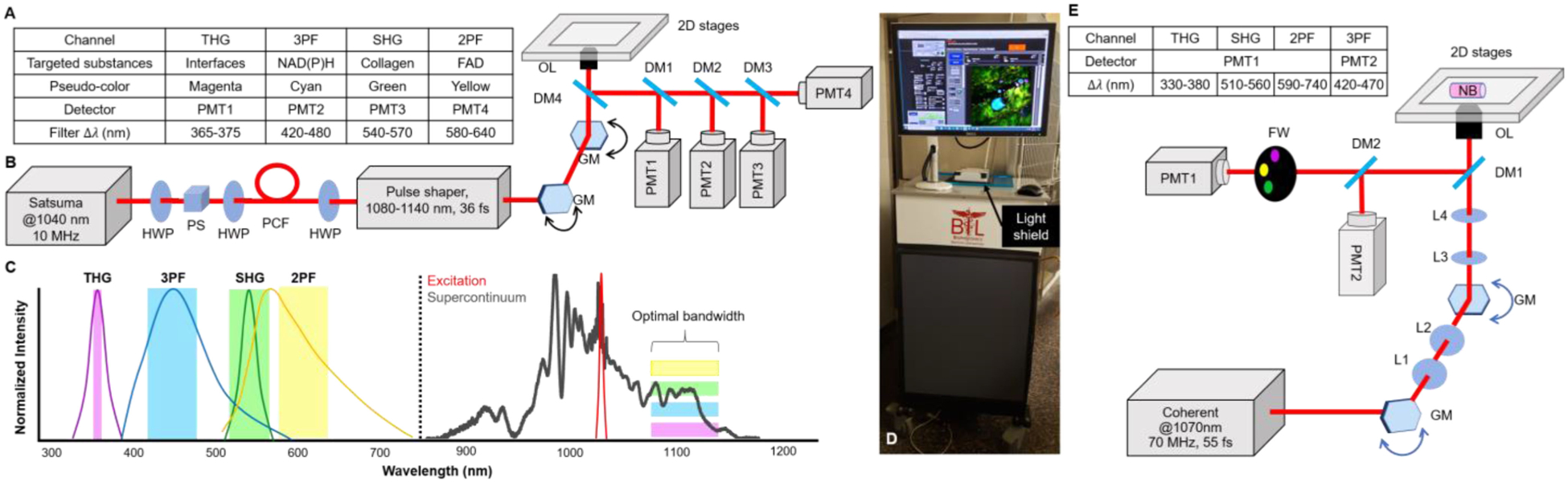
System design comparison between a bench-top SLAM microscope and an intraoperative NLOI system. (A) Table summarizing the color code, corresponding detector, and filtered spectral bandwidth of each individual channel in the SLAM microscope. (B) Optical setup of the SLAM microscope. The system laser source operated at 1040 nm with a 10 MHz repetition rate and was first sent through the photonic crystal fiber (PCF) to generate a supercontinuum (full spectrum shown in (C)). A pulse shaper was programmed to choose the optimal excitation window (1080–1140 nm) and compensate the dispersion for a nearly transform-limited output pulse duration of around 36 fs. Lenses between the galvanometer mirror (GM) scanners and between GM and the objective lens (OL) were omitted for simplification. Different dichroic mirrors (DMs) and optical filters were used before photomultipliers (PMTs) as specified in the table in (A). (C) Normalized spectra and spectral windows for the four different channels listed in the table in (A), excited by the optimal window of the supercontinuum (dark grey) source. (D) Photograph of the portable intraoperative NLOI system with a light shield covering the specimen. (E) Optical setup of the intraoperative NLOI system with a table summarizing differences in the four-channel acquisition. The femtosecond laser centered at 1070 nm excited all four channels simultaneously. PMT1 collects the signals from THG, SHG and 2PF sequentially via filter wheel (FW) switching, while PMT2 simultaneously detects the weaker 3PF signals. The light shield is not drawn in the schematic. HWP, half wave plate; L, lens; NB, needle biopsy specimen; PS, polarization beam splitter; PM, parabolic mirror.

The bench-top SLAM microscope was adapted into a portable multimodal NLOI system and comparisons were first made for the imaging performance using excised human breast tissues acquired during surgical procedures. Figs. 8A, B include a table of the multimodal capabilities as well as a schematic of the SLAM system design, respectively, with four NLOI modalities (THG, 3PF, SHG, and 2PF) excited by a single fiber-based supercontinuum source. We used an industrial fiber laser (Satsuma, Amplitude Systems) to pump a photonic crystal fiber (PCF) (LMA-PM-15) to generate a supercontinuum. The pump laser was centered at 1040 nm, matching the zero-dispersion wavelength of the PCF, which is important for generating low-noise and broad-band supercontinuum [61], [80], [81]. The supercontinuum source was further optimized with a pulse shaper (MIIPS Box640, Biophotonic Solutions Inc.), which performed dispersion compensation to compress the pulse down to around 36 fs, nearly transform-limited. Details of the source are described in our previous work [58], [61]. An optimal excitation window spanning from 1080 nm to 1140 nm was selected for the four NLOI channels (Fig. 8C) [60]. Since THG signal wavelengths are one-third of the excitation wavelengths [53]–[55], long-wavelength excitation becomes essential to avoid strong absorption in biological tissue and optical components [56], [60], which necessitates SLAM excitation to be at least 1080 nm [60]. The emission windows for 2PF and 3PF were selected based on the emission spectra of FAD and NAD(P)H, respectively [44]. The corresponding excitation windows were optimized mainly based on the signal strength and potential for photodamage, which gave the upper limit at around 1140 nm [60]. It is known that the signal intensity is proportional to $\frac{{\langle I\left(t\right)\rangle }^{n}}{{\left(\tau f\right)}^{n-1}}$ [41], [42], [56], where 〈*I*(*t*)〉 is the time-averaged excitation intensity, *n* is the order of nonlinearity (2 for SHG and 2PF, 3 for THG and 3PF), *τ* is the pulse duration, and *f* refers to the pulse repetition rate. Considering that photodamage from multiphoton excitation around 1*μ*m was previously shown to have an order between 2 and 3 [82], this sets a limit on the maximum excitation intensity. Although theoretically, a low pulse repetition rate would be favorable to maximize the signal intensity, in practice it is limited by the resulting slow acquisition speed and saturation (and thus potential damage) of the detectors. In the end, a moderately low pulse repetition rate at 10 MHz was chosen for SLAM microscopy as an optimal trade-off. CARS was not included in the SLAM system since its preferrable excitation wavelengths should be in a range of 780–980 nm [28], where the SLAM supercontinuum source intensity was weak, as shown in the excitation spectrum in Fig. 8C.

For the translation of the lab-based bench-top SLAM system to an intraoperative NLOI system (Fig. 8D), special considerations and trade-offs were made. First, to accommodate the optical setup in a compact and portable cart (35”W × 35”L × 45”H), reductions in the optical source components occurred. Considering that the fiber-based supercontinuum source has a nontrivial operator time and expertise requirement for sensitive optical alignment and frequent maintenance, it was not practical to incorporate this source component into the portable system. Thus, a commercial compact fiber laser (Fidelity-2, Coherent Inc.) was chosen as the excitation source (Fig. 8E). This source delivered sub-55-fs near-transform-limited pulses at a repetition rate of around 70 MHz. Theoretically, the nonlinear signal strength is decreased by 10 times for SHG and 2PF, and decreased 100 times for THG and 3PF, compared to the SLAM microscopy system with the same excitation light intensity, which is the major compromise for the use of this source. In the spectral domain, this laser offered a 70-nm spectral bandwidth centered at 1070 nm, which is similar to the optimal window for SLAM microscopy. The maximum output of the laser was 2 W, which was sufficient for multiphoton excitation. Additionally, this source has built-in dispersion-tunability for users to fine-tune and optimize the output pulse-width for different samples. This feature reduces the need to construct and optimize an external pulse compressor and is flexible and stable. Overall, this laser source benefits the portable system in terms of stability and compactness, but its repetition rate and pulse duration are not ideal, compared to the larger lab-based SLAM microscopy source.

Due to the limited space inside the cart, a 4-PMT simultaneous detection scheme was not possible. Our first-generation intraoperative NLOI system adopted a sequential acquisition scheme by switching four filters using a motorized filter wheel (FW, FW102C, Thorlabs, Inc.) and a single PMT. This reduced acquisition speed by a factor of four compared to SLAM imaging, with the same scanning parameters. To improve this acquisition speed, we further optimized the spatial arrangement of all the mechanical, electrical, and optical components, and were able to add a second PMT for the detection of the weaker 3PF signals, along with the corresponding spectral filter for wavelength-specific detection. Dedicating one PMT for a longer acquisition time was implemented because we found from previous experiments that in most cases the 3PF signals were the weakest among all the channels, and in theory, the 3PF signal is determined by the 5^th^ order nonlinear optical susceptibility and thus the third-order absorption coefficient [53]–[55]. It has been found that the third-order absorption coefficients are often on the order of 10^−20^ cm^2^/W^2^, while the second-order absorption coefficient (for 2PF) is around 10^−10^ cm/W [55].

For imaging, specimens were placed on a glass window that was flush with the top surface of this portable system, with an inverted microscope objective and scanning system within the cart. In this configuration, a light concealment design was needed, which included a light-shield box on the top of the cart, covering the specimen. This prevented exposure of the PMTs to ambient light which would add significant background noise and thus degrade image quality. Furthermore, with 2D translation stages installed on this system to enable scout-imaging and mosaicking of the NB core specimens, more careful light-tight considerations were made to ensure that with the moving sample holder on top of the stages, minimal light would penetrate the cart and system. The complete design of the system is shown in Figs. 8D, E. After the high-power laser source, a variable attenuator (NDC-100C-4M, Thorlabs Inc.) was placed at the output to ensure the average power incident on the biological sample was around 20 mW. Laser beam scanning was achieved by a pair of galvanometer-scanning mirrors (62xxH Series Galvanometer XY Sets, Cambridge Technology, Inc.) with a pair of relay lenses in between to form a 4-f relay system. After the galvo-scanning mirrors, there was another 4-f system (lens pair shown as blue ellipses in Fig. 8E) before reaching the objective (OL, XLPLN25XWMP2, Olympus, Inc.) which had a numerical aperture of 1.05 and a magnification of 25X. The objective was mounted on a linear piezo stage (SLC-24120, SmarAct GmbH) for fine adjustment of its axial position. Before the detection PMTs, dichroic mirrors were chosen to reject laser light reflected and scattered from the specimen, while permitting the transmission of the nonlinear signals. Two photomultiplier tubes (H7421-40, Hamamatsu Photonics) were implemented to detect the nonlinear signals. For each modality, a spectral band-pass filter was chosen according to the theoretically estimated values calculated from the excitation wavelength (1070 nm) [53] and previously reported studies [44], [46] (Table in Fig. 8A). The two-photon excitation spectrum of FAD [44] and the three-photon excitation spectrum of NAD(P)H [46] were considered for the estimated emission peak wavelengths for 2PF and 3PF, respectively. Although ideally there should be no spectral overlapping among all four channels, as illustrated in Fig. 8E, in practice, strong SHG and 2PF signals may leak into adjacent channels, because of the spectral overlap of the SHG spectrum and the FAD emission spectrum, as shown in Fig. 8C. Thus, it was important to ensure that none of the channels were saturated during imaging.

**Fig. 9.**
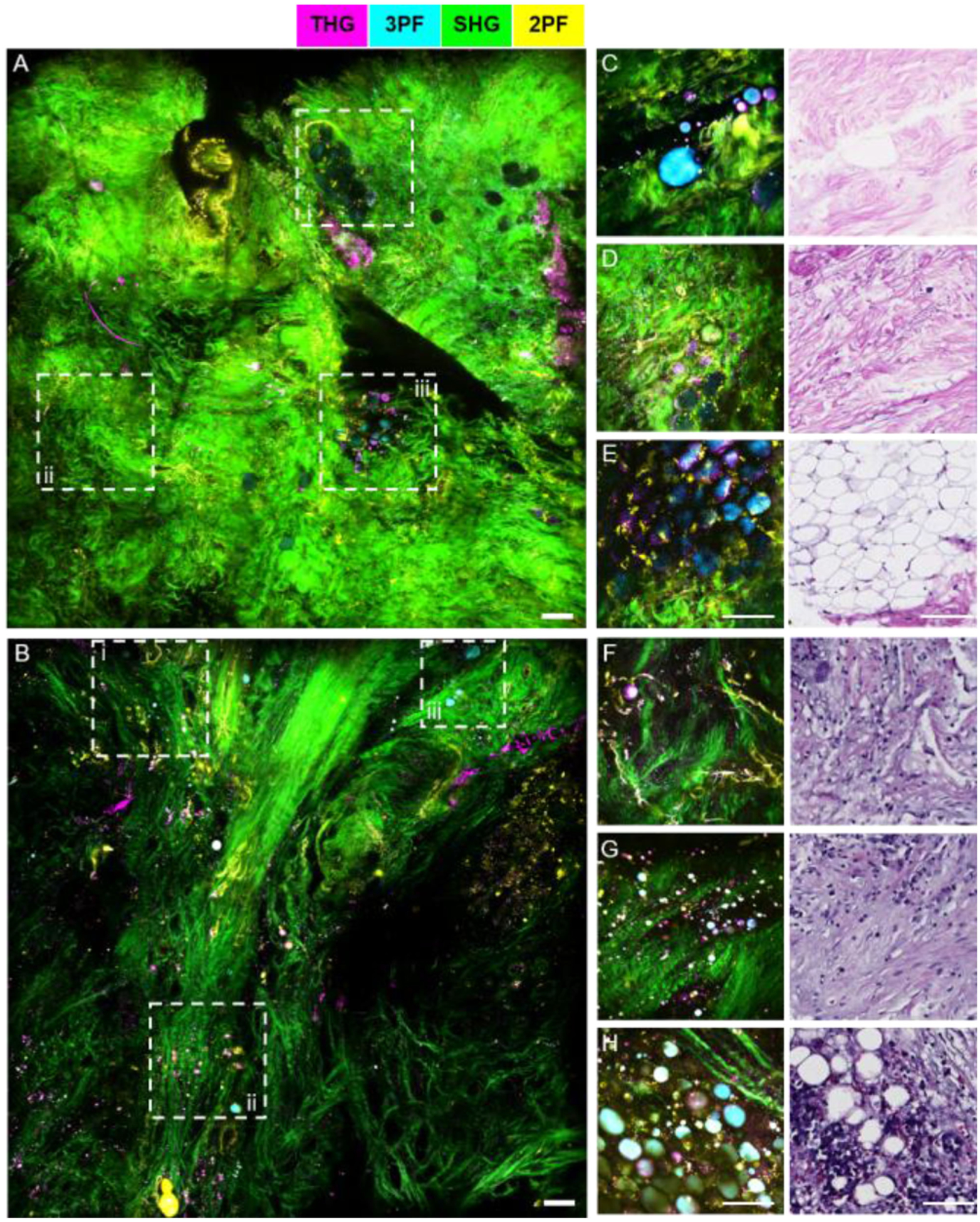
Comparison between SLAM microscopy and intraoperative NLOI systems for imaging excised human breast tissue from the same patient. (A)–(B) Large FOV SLAM images of (A) adjacent normal tissue and (B) tumor tissue excised from a breast cancer patient diagnosed with IDC. (C)–(E) images of adjacent normal breast tissue from the same patient acquired by the intraoperative NLOI system (left) and correlated with histology results (right). (F)–(H) Images of tumor tissue from the same human breast cancer patient acquired by the intraoperative NLOI system (left) and correlated with histology results (right). The dashed boxes (i–iii) in the SLAM images (A), (B) highlight features which can also be identified in the intraoperative NLOI results (C)–(E), (F)–(H). Color scale is shown on the top of the Figure. Scale bar represents 100 *μ*m.

The intraoperative NLOI system was also designed to have completely automated control of all electrical and mechanical components, such as the data acquisition (DAQ) card (PXI-6329, National Instrument), 2D translational stages, 1D axial positioning stage, and the filter wheel. All the automated controls were integrated into a single LABVIEW program with a simple user interface (Fig. 8D). As a result, this new intraoperative system was able to acquire 4-channel multimodal images with sub-micron pixel resolution in around 1.5-minutes, with each 500 *×* 500 pixels frame having a FOV of 350 *μ*m × 350 *μ*m. For these images, the sampling rate was 10 kS/s, which is lower than the full capability (16-bit, 250 kS/s) of the DAQ card. The major limitation during acquisition is the SNR. Among all four channels, 3PF is usually the weakest due to its highest order of nonlinearity. The lateral resolution of the system was measured to be around 557 nm by imaging 100-nm diameter fluorescent beads, and the maximum single FOV was measured to be around 550 *μ*m *×* 550 *μ*m by imaging a reflective grid target. The axial resolution was estimated to be 2.8 *μ*m by fitting the intensity profiles of 1-*μ*m fluorescent beads versus the axial position of the objective. We compared the imaging performance of the newly up-graded intraoperative label-free multimodal NLOI system with the current SLAM system by imaging human breast tissue excised from the same patient (Fig. 9). The received tissue specimen was divided in half, with each half being imaged by one of the two systems. The larger FOV of the SLAM images of adjacent normal (Fig. 9A) and IDC (Fig. 9B) tissue were obtained by translational mosaicking. Each 4-modality set of SLAM images took 2 s to acquire, and consisted of 900 × 900 pixels, 450 *μ*m *×* 450 *μ*m FOV for each image. Images acquired from the adjacent normal tissue by the intraoperative NLOI system (Fig. 9 C–E) show features consistent with curly collagen fibers (highlighted by SHG in green), elastin fibers (highlighted by 2PF in yellow) and densely packed adipocytes (highlighted by 3PF in cyan), which are verified by the corresponding histology images. Similar features were found in the large FOV SLAM image (Fig. 9A). For example, Fig. 9A(iii) shows adipocytes embedded in the dense stroma, which are also shown in Fig. 9(C–E). Fig. 9A(ii) shows curly collagen fibers and elastin fibers, which can also be found in Fig. 9D. While the SLAM image captured a FOV of 1.85 *×* 1.85 mm^2^, showing different features in different regions (Fig. 9B), the images captured by the intraoperative NLOI system were randomly sampled around the tumor site to visualize additional features of the tumor microenvironment, such as nested collagen fibers (Fig. 9F), aligned collagen fibers with tumor cells (Fig. 9G), and adipocytes (Fig. 9H) interspersed along with the overall orientation of the collagen fibers. These features are also visible in the SLAM image (Fig. 9B, i–iii) and verified by the corresponding histology. Based on these results, the intraoperative NLOI system offers comparable imaging performance for detecting biomarkers that can be used for visual and AI/DL-automated cancer detection and diagnosis.

## V. Point-of-Procedure Assessment of Needle Biopsies Using Label-Free Multimodal Nonlinear Optical Imaging

**Fig. 10.**
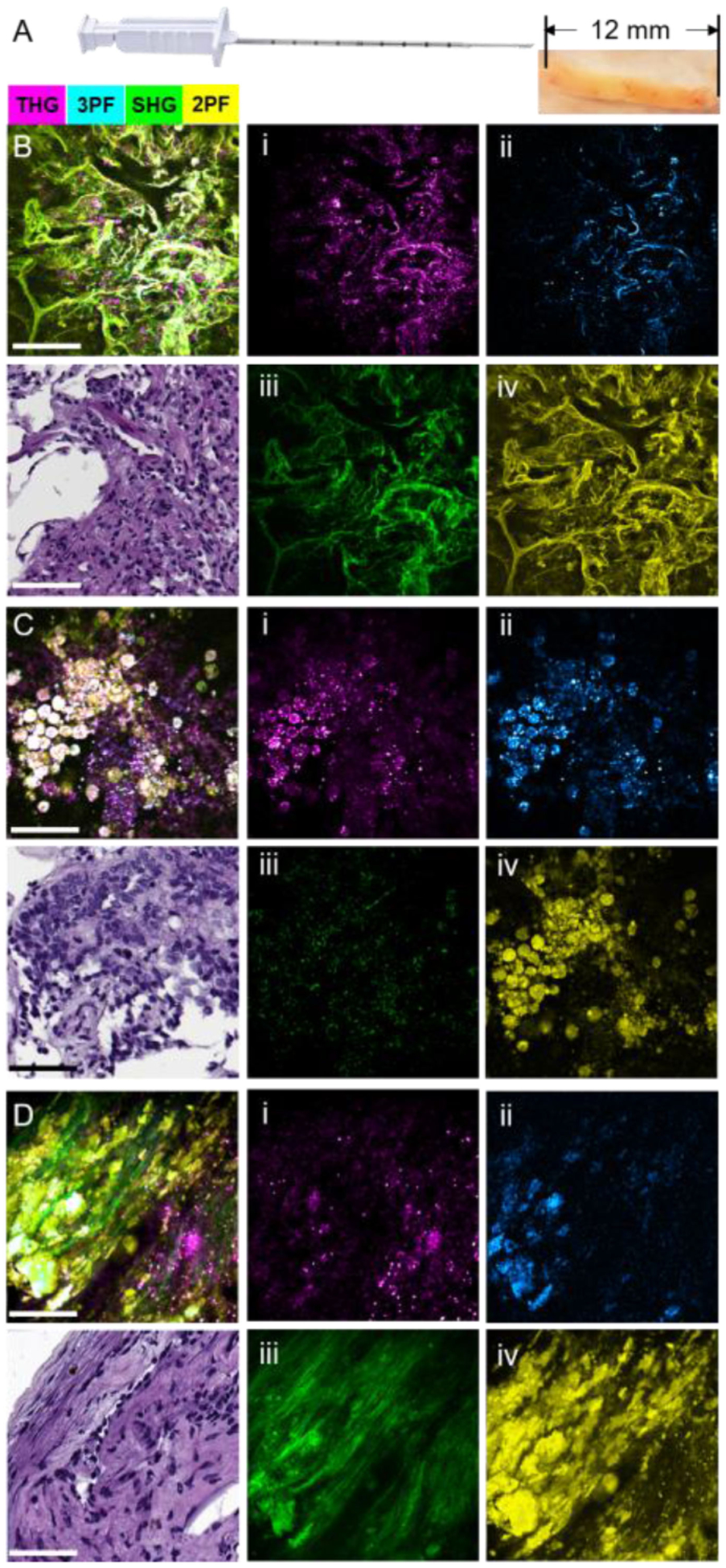
Label-free multimodal NLOI images of core NB specimens. (A) Photograph of the core NB device and representative NB specimen. NB core specimens were around 1.6-mm in diameter and 12-mm in length. (B)–(D) Intraoperative multimodal NLOI results of the tumor microenvironment from NB core specimens acquired from a canine pulmonary adenocarcinoma, showing the composite (merged) image and corresponding H&E histology, as well as each of the 4 modality channels shown separately at the right side (i)–(iv). (B) Image set from a core NB specimen acquired *in vivo* from a visually normal region adjacent to the tumor. (C) Image set from a core NB specimen acquired *in vivo* from the tumor. (D) Image set from a core NB specimen acquired *ex vivo* from the freshly excised lung tumor. (i) THG, magenta; (ii) 3PF, cyan; (iii) SHG, green; (iv) 2PF, yellow. Color scale is given on the top of the Figure. Scale bar represents 100 *μ*m.

To further demonstrate the real-time point-of-care imaging and diagnostic capabilities of our portable system, core NB specimens obtained during canine companion animal surgeries were evaluated with label-free multimodal NLOI. Core NB specimens were taken *in vivo* by a veterinary surgical oncologist from several tissue regions before surgical resection of the larger tissue/organ mass, including the tumor and the normal adjacent sites. Immediately following NB, the specimens were imaged by the intraoperative multimodal NLOI system while the surgeon proceeded with the larger tumor resection. Here the *in vivo* extraction of biopsies emphasizes that the biopsies are taken from the intact tumor or organ from the live animal in contrast to extraction from an *ex vivo* mass after removal. Immediately after the larger tumor mass was removed, additional core NB specimens were acquired from the *ex vivo* tumor mass and imaged. The NB device was a 14-gauge manual Tru-cut needle (Merit Medical Inc.), which can acquire NB cores of around 1.6-mm diameter and around 12-mm in length (Fig. 10A). The overall length of the NB specimens varied depending on the mechanical integrity of different tissue types and techniques of different surgeons.

Intraoperative multimodal NLOI results from a canine pulmonary adenocarcinoma NB specimen are shown in Fig. 10(B–D), along with corresponding histology results. Individual nonlinear imaging modality channels are shown to the right side of each composite image and corresponding H&E-stained histology section, with THG (i) colored in magenta, 3PF (ii) in cyan, SHG (iii) in green and 2PF (iv) in yellow. The NB specimen from the adjacent normal lung tissue (Fig. 10B) contains normal pneumocytes along the boundary of small airways and alveoli (left bottom hollow region), as well as fibroblasts and macrophages scattered throughout the collagen-rich (SHG, green) connective tissue. In contrast, the image taken from the NB tumor specimen acquired from the *in vivo* organ (Fig. 10C) was dominated by clustered tumor cells (the color saturation is due to the overlapping of the yellow (2PF), cyan (3PF), and magenta (THG) channels). Interestingly, the image of the NB tumor specimen acquired from the *ex vivo* organ shows cell clusters embedded within the dense collagen fibers. This is likely because the NB cores were taken from different locations in the tumor, highlighting the high degree of heterogeneity present. However, this also demonstrates that the information provided by the NB cores acquired *ex vivo* at later times may differ from that provided by the NB cores acquired from the *in vivo* organ, due to the complex and dynamic structural and biochemical processes that are changing over time. Future studies are needed to investigate these associated changes and determine what *in vivo* label-free optical biomarkers persist over time. These results also further exemplify the significance and need for an intraoperative or point-of-procedure evaluation method for NB procedures.

**Fig. 11.**
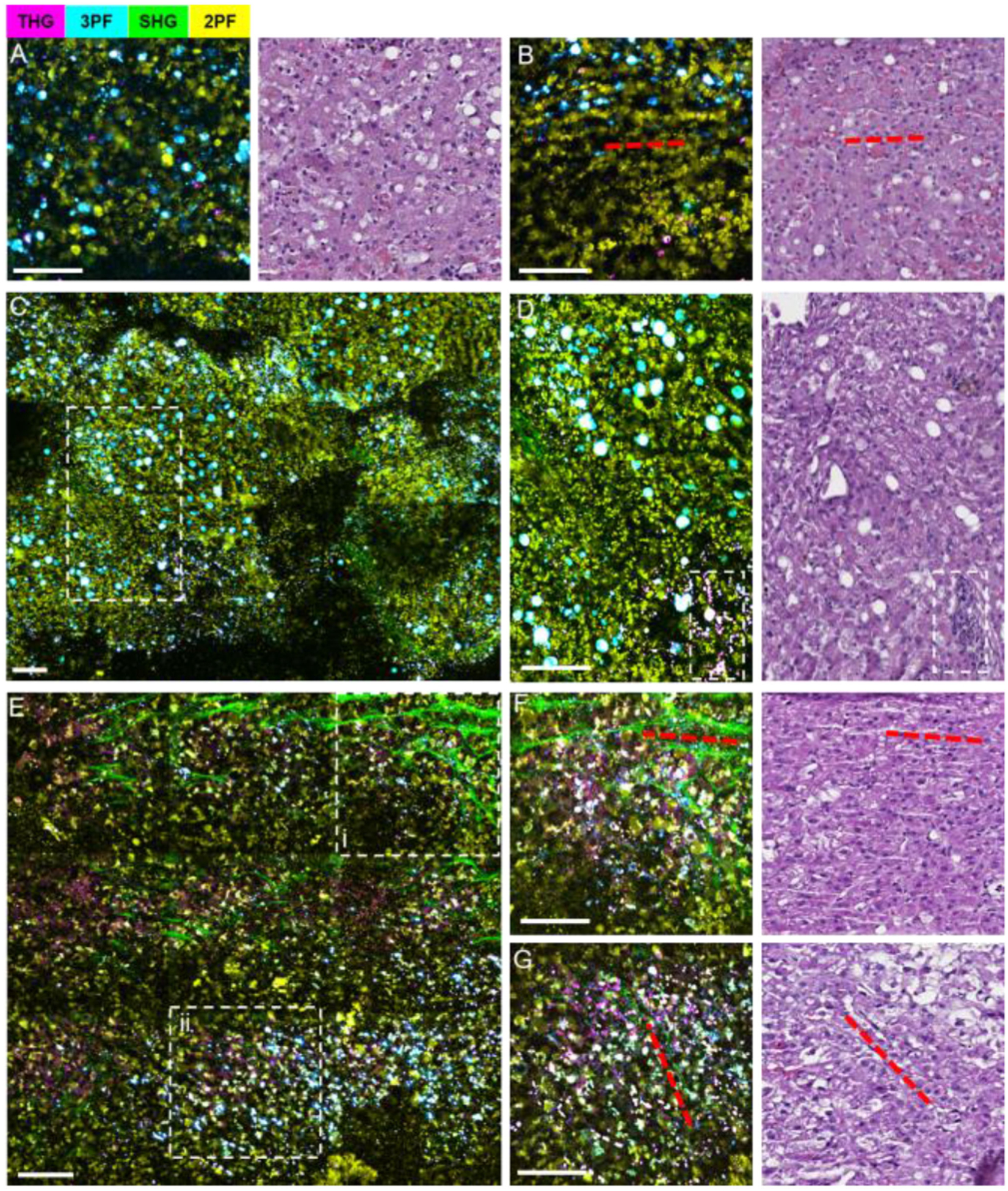
Label-free intraoperative multimodal NLOI imaging of canine liver NB cores with corresponding histology. (A), (B) Normal liver tissue biopsied adjacent to the tumor, showing sparsely distributed hepatocytes, adipocytes, and sinusoids (aligned with red dashed lines). (C) A mosaicked NLOI image from the tumor margin, with an enlarged region (dashed white box) shown in (D) where the incomplete destruction of sinusoids with fibrotic stroma content, adipocytes, and high-density foci of tumor cells (dashed box) are observed. (E) A mosaicked NLOI image of the liver tumor with regions (i, ii) enlarged in (F), (G), respectively. Red dashed lines in (F), (G) and the corresponding histology indicate general collagen fiber orientation, with dense tumor cell infiltration. Color scale is given on the top of the Figure. Scale bar represents 100 *μ*m.

As a second example, Fig. 11 provides label-free multimodal intraoperative imaging results of NB specimens from a canine liver. Fig. 11(A, B) visualizes the microenvironment of the normal-appearing liver taken from a region adjacent to a hepatocellular carcinoma (HCC) where hepatocytes and adipocytes are scattered sparsely, and sinusoids lined with endothelial cells are evident (red dashed line). Fig. 11C provides a larger FOV of the tumor margin within the NB core, with the region contained within the white dashed box enlarged in Fig. 11D. Compared to Fig. 11(A, B) along this tumor margin, an increase in collagen content and cell density can be observed. In Fig. 11D, incomplete destruction of sinusoids was observed as a signature for HCC development, and the dashed white box highlights cell clusters. In contrast, Fig. 11E shows the tumor microenvironment from the NB core taken from the liver tumor. The two dashed white boxes (i, ii) are enlarged in Figs. 11(F, G), respectively, where red dashed lines show general collagen fiber orientations. Compared to Fig. 11D, the collagen fibers are more nested than aligned or bundled, with significant infiltration of tumor cells highlighted by the higher intensity THG and 3PF signals. The ability to visualize a large FOV is beneficial for optical assessment of the tissue microenvironment. Additionally, sparsely patterned sampling over the entire NB core specimen could provide a “scout” image to give a broader view about the specimen, before suspicious areas are targeted for the acquisition of image mosaics.

The use of label-free multimodal NLOI permits point-of-procedure assessment of tissue specimens, relying on contrast from a variety of structural, molecular, and metabolic features. While this latest system has revealed new potential biomarkers and potentially diagnostic information, a few limitations have been identified. First, the acquisition speed of this system is mainly limited by the signal strength of each modality. As stated in the previous section, the high repetition rate of the laser source used in this portable system is not favorable for optimal nonlinear signal generation, although the dispersion tunability of the laser can help ensure the optimum ultrashort pulse width at the sample plane. At this time, there is no commercially available compact source with comparable performance, and a customized laser source may not be sufficiently stable and turn-key for intraoperative imaging. Second, because of the limited space in the cart, simultaneous acquisition of all four (or more) nonlinear modality signals (as in SLAM microscopy) was not feasible. The 2D translational stages embedded inside the cart also have a limited movable range. The highly sealed design also causes issues with heat dissipation which may risk malfunction of electronic components after long-term continuous use. Despite implementing a mesh bottom with circulation fans, significant heat reduction was not observed, and future designs must consider trade-offs between compactness, system components, cart construction, and heat dissipation. Although the current system is optimized for tissue imaging, imaging of fluid samples, such as from fine-needle aspirates or liquid biopsies, is possible with a customized holder. Despite these limitations, this intraoperative multimodal NLOI system is able to provide point-of-procedure optical assessment of fresh unstained biological specimens, whether from NB procedures or intraoperative surgical resections, and in combination with customized AI/DL algorithms, offers great potential to assist in real-time cancer diagnosis. Future improvements on this intraoperative multimodal NLOI system may include the replacement of the current high-rep-rate laser and addition of two more PMTs to enable completely simultaneous detection. We are also working on fiber-based intraoperative SLAM systems for applications in endoscopy and with handheld probes.

## VI. Conclusion

This paper presented a brief review of the technical developments and demonstrations that have led to an intraoperative label-free multimodal NLOI technique that integrates SHG, THG, 2PF, and 3PF to provide microstructural, biomolecular, and metabolic information from unperturbed tissue and tumor microenvironments. This technique has been adapted and translated into a portable intraoperative NLOI system that has been previously demonstrated in human breast cancer surgeries. With recent system upgrades, the capability for rapidly imaging fresh, unstained core NB specimens acquired from both *in vivo* and *ex vivo* tumors and adjacent normal tissues during veterinary canine surgeries was demonstrated. We believe that label-free multimodal NLOI can be used as a powerful tool to assist in the intraoperative assessment of the tissue and tumor microenvironment, including the identification of tumor-associated EVs as new biomarkers for cancer detection and staging. Further, label-free multimodal NLOI can be used for the real-time screening of abnormal tissue within NB specimens as well as in human or AI-assisted cancer diagnosis at the point-of-procedure, when such clinically useful information is needed most.
